# ﻿Four new species of the genus *Luzonomyza* Malloch (Diptera, Lauxaniidae) from China

**DOI:** 10.3897/zookeys.1074.68392

**Published:** 2021-12-01

**Authors:** Wenliang Li1, Xulong Chen1, Keli Feng1, Shengjuan Zhao2, Ding Yang3

**Affiliations:** 1 College of Horticulture and Plant Protection, Henan University of Science and Technology, Luoyang 471023, China; 2 College of Food & Bioengineering, Henan University of Science and Technology, Luoyang 471023, China; 3 Department of Entomology, China Agricultural University, Beijing 100193, China

**Keywords:** Lauxaniinae, Lauxanioidea, new species, Oriental region, Schizophora

## Abstract

Four species of the genus *Luzonomyza* Malloch, 1929 from southwest China are described as new to science: *Luzonomyzavittifacies* Li & Yang, **sp. nov.**, *L.serrata* Li & Yang, **sp. nov.**, *L.honghensis* Li & Yang, **sp. nov.**, and *L.brevis* Li & Yang, **sp. nov.** A key to *Luzonomyza* species is also presented.

## ﻿Introduction

The genus Luzonomyza Malloch, 1929 was erected as a subgenus of Trigonometopus Macquart 1835 on the basis of a single species from the Philippines, *Trigonometopusbakeri* Bezzi, 1913. From the 20^th^ to the 21^st^ century, considerable contributions were made to the genus by several taxonomists. In his key, Stuckenberg (1971) first recognized *Luzonomyza* (= *Luzonomyia*) as a valid subgenus. Shatalkin (1997) described a new species Trigonometopus (Tetroxyrhina) nigripalpis from Thailand (at that time, TetroxyrhinaHendel, 1938 was also considered a subgenus of Trigonometopus), and later, he described another new species, *L.sinica* Shatalkin, 1998, from China and Thailand. A new species (*L.japonica*) was described from Japan in *Trigonometopus*, and the male genitalia was figured (Sasakawa 2002). Papp (2007) described three new species, *L.pseudoforficula*, *L.sasakawai*, and *L.vietnamensis*, providing several figures (the epandrium of *L.bakeri*, the wing and male genitalia of *L.sinica* and *L.pseudoforficula*, and the head of *L.sasakawai* and *L.vietnamensis*) and presented a key to the Old World genera of Trigonometopini, including *Luzonomyza* and a key to the species of the genus *Luzonomyza*. Shi and Yang (2015) also described two new species and presented a key to species of the genus. To date, 13 species of *Luzonomyza* are known in the world, and all are distributed in the Oriental Region. Seven species occur in China, including four new species from southwest China described in this paper.

*Luzonomyza* can be recognized by the combination of the following features: head triangular, frons projecting beyond eye, usually with brown longitudinal band and with short setulae on anterior half; ocellar setae short. Fronto-facial angle acute. Face long, inclinate, some species with distinct genual spine, parafacial with a row of short setulae on lower half along inner margin. Gena with a row of long setae extending from middle to lower half of parafacial, but not in line with setulae on inner margin. Mesonotum with wide brown band; three post-sutural dorsocentral setae; katepisternum with a seta. Wing hyaline, anteriorly usually pale brown to dark brown, crossveins r-m and dm-cu with brown spots. Abdomen yellow except posterior margin of tergites usually brown or black. (Malloch 1929; Stuckenberg 1971; Papp 2007; Shi and Yang 2015).

## ﻿Materials and methods

### ﻿Material

All specimens were collected in Yunnan Province, China. The specimens of *L.brevis*, *L.honghensis*, and *L.serrata* were captured alive and fixed in 75% ethanol. The specimen of *L.vittifacies* was killed with ethyl acetate and dried for morphological examination. All specimens are deposited at the China Agricultural University, Beijing, China (**CAUC**).

### ﻿Morphological descriptions

General terminology follows Cumming and Wood (2009) and Gaimari and Silva (2010). Genitalia preparations were made by removing and macerating the apical portion of the abdomen in cold saturated NaOH for 6 h, after which they were rinsed and neutralized for dissection and study. After examination in glycerin, they were transferred to fresh glycerin and stored in a microvial pinned below the specimen or moved to ethanol in a tube together with the wet specimens.

## ﻿Taxonomy

### ﻿Key to the known species of the genus *Luzonomyza* Malloch

(modified from Papp 2007 and Shi and Yang 2015)

**Table:** 

1	All tarsi entirely yellow	***L.vietnamensis* Papp**
–	At least a pair of tarsi apices brown to black	**2**
2	Male surstylus with broad, slightly bifid apex, which is concave in lateral view	***L.bakeri* (Bezzi)**
–	Male surstylus not bifid apex in lateral view	**3**
3	Wing unicolorous	**4**
–	Wing patterned	**5**
4	Wing hyaline, very faintly yellow-tinged along anterior margin; haltere yellow with faintly brown-tinged knob; male epandrium without dorsoapical processes; postgonites simple and long	***L.japonica* (Sasakawa)**
–	Wing unicolorous brown; knob of haltere rather dark; male epandrium with extremely long dorsoapical processes, postgonites short with characteristic apex	***L.pseudoforficula* Papp**
5	Palpus black	***L.nigripalpis* (Shatalkin)**
–	Palpus yellow	**6**
6	Male genitalia with epandrium lacking dorsoapical processes	***L.sasakawai* Papp**
–	Male genitalia with epandrium having strong dorsoapical processes	**7**
7	Mesonotum with 3 brown stripes	***L.sinica* Shatalkin**
–	Mesonotum with 4 brown stripes	**8**
8	Frons with 3 brown longitudinal stripes, median longitudinal stripe wide extending from anterior margin to ocellar triangle, lateral longitudinal stripes narrow	***L.brevis* sp. nov.**
–	Frons with a brown or blackish brown longitudinal stripe, extending from anterior margin to ocellar triangle	**9**
9	Acrostichal setulae in 2 rows	**10**
–	Acrostichal setulae in 4 rows	**11**
10	Hind femur without long anteroventral seta; epandrium with a pair of long black coniform dorsal processes, surstylus tapering distally and incurved	***L.gaimarii* Shi & Yang**
–	Hind femur with a long anteroventral seta; epandrium with a pair of short black coniform dorsal processes, surstylus bluntly rounded, not incurved distally	***L.hirsute* Shi & Yang**
11	Face without brown stripes	***L.honghensis* sp. nov.**
–	Face with 2 brown stripes	**12**
12	Legs yellow, fore tarsomeres 4 and 5 brown; abdomen yellow, tergites 2–6 black on posterior margin, but tergite 6 yellow posteriomedially; hypandrium broad, membranous; pregonite confluent with hypandrium; phallus with a pair of lateral acute processes near base in ventral view, concavity V-shaped, with a dorsal acute process subapically in lateral view, apex acute and curved dorsally	***L.vittifacies* sp. nov.**
–	Legs pale yellow, fore femur and basal half of tibia brown, tarsomeres 4 and 5 pale brown, mid and hind femora and tibiae yellow but pale yellow at apex; abdomen yellow, tergites 2–6 blackish brown on posterior margin; hypandrium V-shaped, with 2 pairs of lateral acute processes; pregonite indistinct, hypandrium, pregonite and phallus confluent together; both sides of phallus serrated, with a dorsal process at apex in lateral view	***L.serrata* sp. nov.**

### ﻿Species descriptions

#### ﻿ Luzonomyza brevis

Taxon classificationAnimaliaDipteraLauxaniidae

Li & Yang sp. nov.

C0B0660D-6F21-5D03-9325-5A38E4807138

http://zoobank.org/55D839C9-A2BD-4545-976B-C7C0B3F989F2

Figures 1–4 , 5–8

##### Type material.

***Holotype*.** ♂ (CAUC), China, Yunnan Province, Longling County, Xiaoheishan Reserve; 24°33'51"N, 98°49'20"E; 1840 m; 26 Apr 2012; Wenliang Li leg.

**Figures 1–4.**
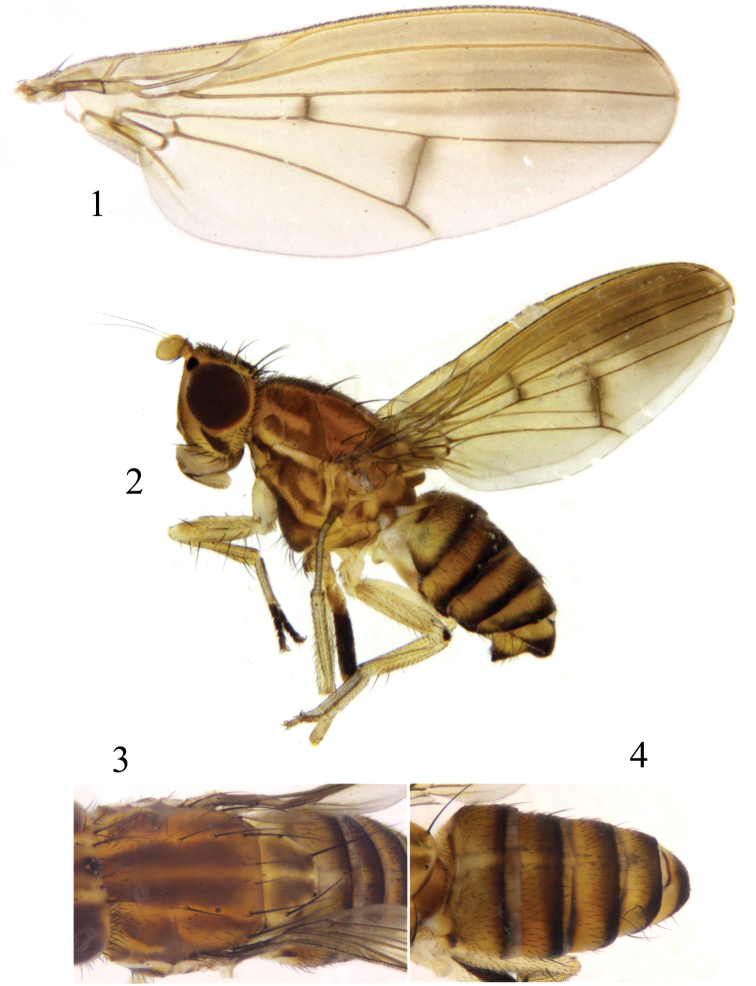
*Luzonomyzabrevis* sp. nov. Male **1** wing **2** habitus, lateral view **3** thorax, dorsal view **4** abdomen, dorsal view.

##### Etymology.

Latin, *brevis*, referring to the epandrium with short dorsoapical processes. It is an adjective in apposition.

**Figures 5–8.**
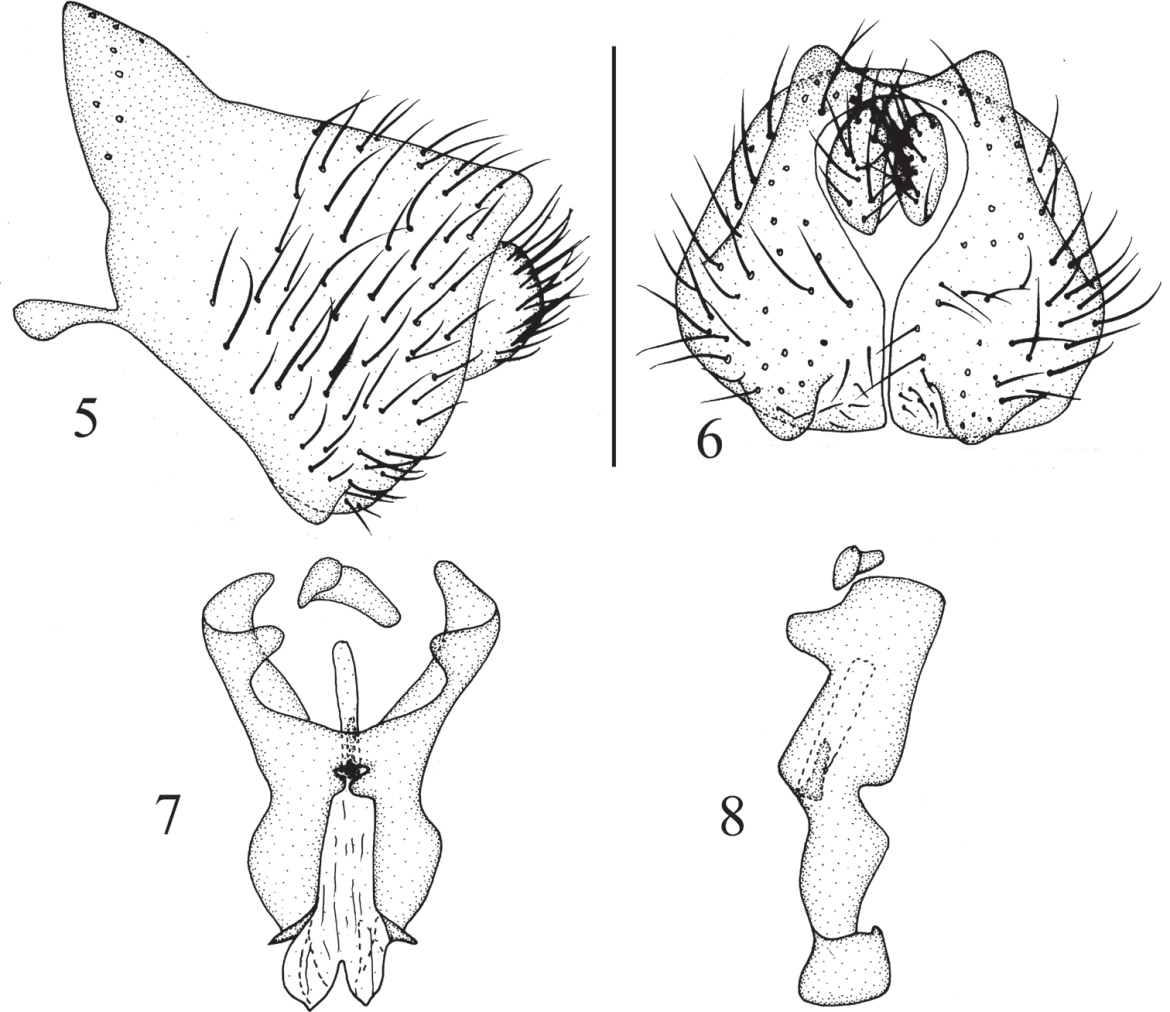
*Luzonomyzabrevis* sp. nov. Male **5** syntergosternite and epandrium, lateral view **6** epandrial complex, posterior view **7** phallic complex, ventral view **8** phallic complex, lateral view. Scale bar: 0.5 mm.

##### Diagnosis.

Frons with 3 brown longitudinal stripes, median longitudinal stripe wide, extending from anterior margin to ocellar triangle, lateral longitudinal stripes narrow; face with a diamond-shaped brown marking in the middle, four angles of marking extending to facial margins. Mesonotum with 4 brown stripes. Epandrium with paired dorsoapical processes; phallapodeme claviform, shorter than phallus.

##### Description.

**Male.** Body length 4.8 mm, wing length 4.3 mm.

***Head*** yellow. Face with an angular hump on middle of basal half, lateral margin brown on apical half, with a diamond-shaped brown marking in the middle, 4 angles of marking extending to facial margins; parafacial with sparse short hairs, with a black spot between eye and antennal bases, and with 5 long setae extending to gena. Frons ~1.2× longer than wide and parallel-sided, with 3 brown longitudinal stripes, a broader brown median longitudinal stripe extending from anterior margin to ocellar triangle, 2 lateral longitudinal stripes narrower and pale brown, anterior half with short setulae; ocellar triangle grayish black, ocellar setae very small, hair-like, anterior fronto-orbital seta reclinate, shorter than the posterior one. Gena with broad brown stripe, ~1/3 height of eye. Antenna yellow, rounded apically, ~1.2× longer than high; arista brown except for yellow base, pubescent. Proboscis yellow with white and black setulae, and with a pair of irregular blackish-brown lateral spots apically; palpus yellow with black setulae.

***Thorax*** (Fig. 3) brownish yellow, with grayish-white pruinescence. Mesonotum with 4 brown stripes extending to tip of scutellum, occupying most of scutellum. 0+3 dorsocentral setae, anteriormost dorsocentral seta away from scutal suture; acrostichal setulae in 4 rows; a pair of prescutellar setae, shorter than anteriormost dorsocentral seta. Dorsal and posterior margin of anepisternum and dorsal margin of katepisternum pale yellow. One anepisternal seta, 1 katepisternal seta. ***Legs*** mostly yellow; fore tarsomeres 2–5 pale brown, mid-legs differently colored: only right mid femur blackish brown but brownish yellow at base. Fore femur with 7 posterodorsal setae and 6 posteroventral setae, fore tibia with a long dorsal preapical seta and a short apicoventral seta. Mid tibia with a strong dorsal preapical seta and an apicoventral seta. Hind femur with a weak preapical anterodorsal seta; hind tibia with a long dorsal preapical seta and a short apicoventral seta. ***Wing*** pale brown along costal margin, extending to M_1_, a brown spot on each of the crossveins r-m and dm-cu; subcostal cell brown; costa with 2^nd^ (between R_1_ and R_2+3_), 3^rd^ (between R_2+3_ and R_4+5_) and 4^th^ (between R_4+5_ and M_1_) sections in ratios of 9.7: 1.5: 1.4; r-m on middle of discal cell; ultimate and penultimate sections of M_1_ in ratios of 6.6: 3.5; ultimate section of CuA_1_ ~1/5 of penultimate. Haltere pale yellow.

***Abdomen*** (Fig. 4) yellow, tergites 2–6 blackish brown on posterior margin. ***Male genitalia*** (Figs 5–8): syntergosternite confluent with epandrium, near triangular. Epandrium with a pair of long conical dorsoapical processes in lateral view, a pair of lateral processes broad apically and narrow basally on anterior margin. Surstylus situated in ventral angle and small; hypandrium V-shaped, with 2 pairs of inner apical processes. Gonopod absent, hypandrium confluent with phallus. Phallus with a pair of lateral preapical acute processes in ventral view; dorsal process broader in lateral view, vertical to phallus, anterior apical angle acute. Phallus deeply concave apically, phallapodeme claviform, shorter than phallus.

**Female.** Unknown.

##### Remarks.

This new species is very similar to *Luzonomyzasasakawai* from Thailand and Vietnam by the body markings and wing type, but it can be separated from the latter by the 3 narrow brown stripes on the frons; by the brown fore tarsomeres 2–5; by the epandrium with dorsoapical processes. In *Luzonomyzasasakawai*, the frons has a narrow brown stripe; the fore tarsomeres 3–5 are black; the epandrium is without dorsoapical process.

##### Distribution.

China (Yunnan).

#### ﻿ Luzonomyza honghensis

Taxon classificationAnimaliaDipteraLauxaniidae

Li & Yang sp. nov.

9DE3DF8A-8ED8-50B9-A052-8E43F1A4012C

http://zoobank.org/D8D043A7-C691-4830-9AFA-8BDC6625CAB3

Figures 9–12 , 13–16

##### Type material.

***Holotype*.** ♂ (CAUC), China, Yunnan Province, Honghe Hani and Yi Autonomous Prefecture, Qimaba township; 22°48'33"N, 102°14'31"E; 1010 m; 11 Jun 2013; Jinying Yang leg.

**Figures 9–12.**
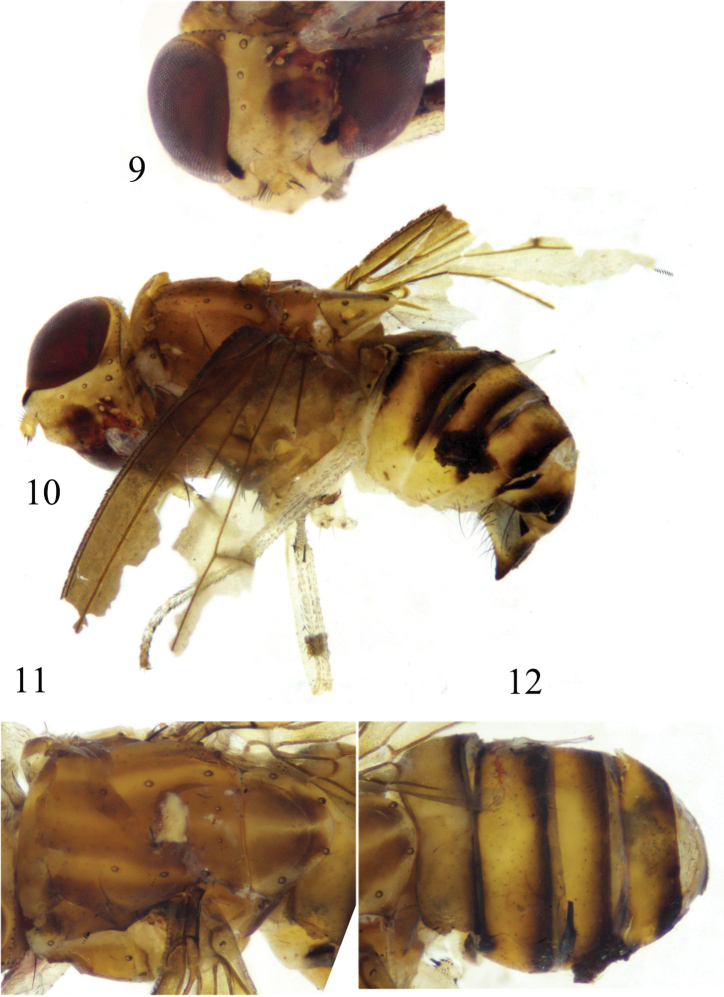
*Luzonomyzahonghensis* sp. nov. Male **9** head, anterior view **10** habitus, lateral view **11** thorax, dorsal view **12** abdomen, dorsal view.

##### Etymology.

The specific name refers to the holotype locality, Honghe Prefecture. It is a noun in genitive case.

**Figures 13–16.**
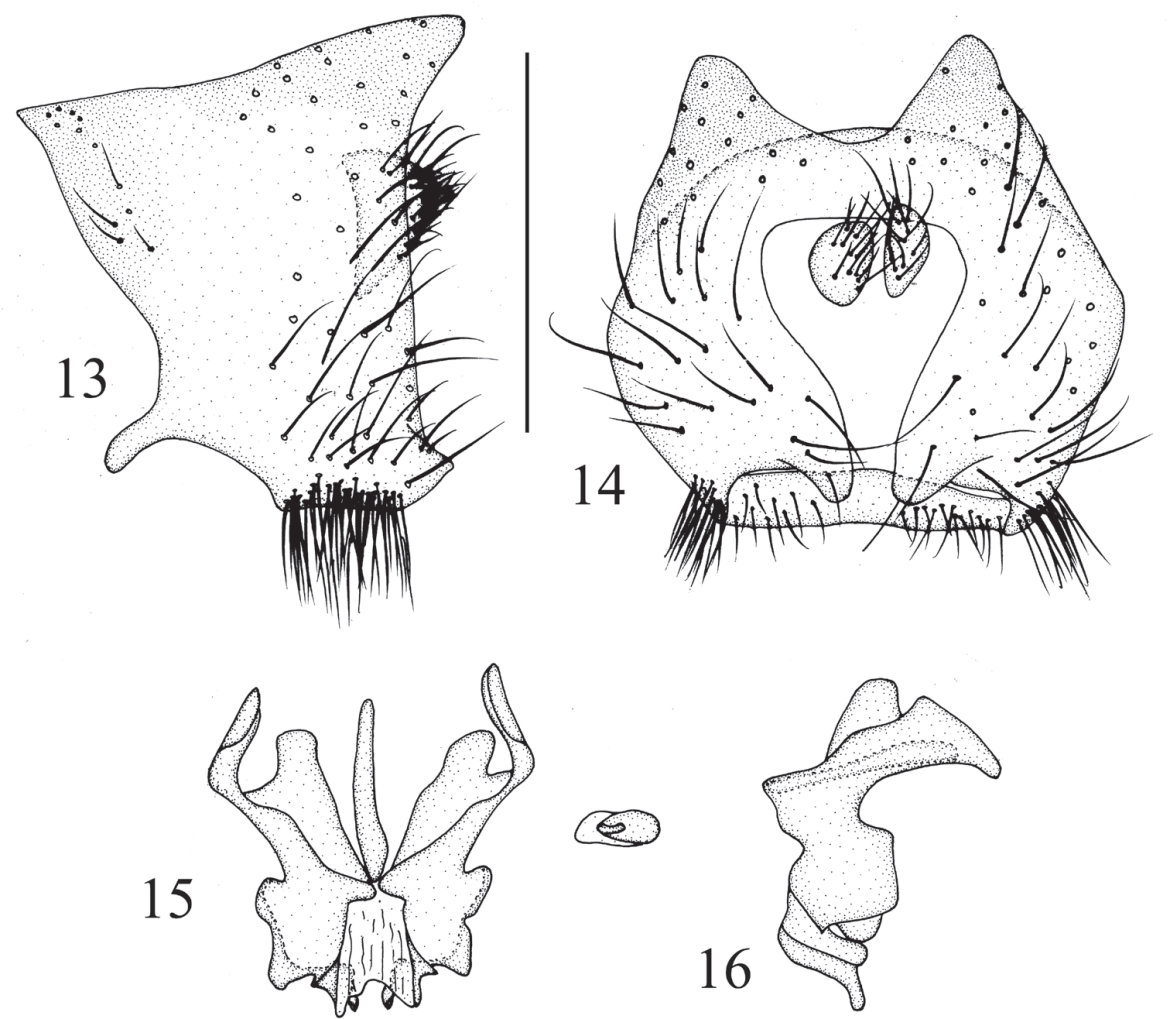
*Luzonomyzahonghensis* sp. nov. Male **13** syntergosternite and epandrium, lateral view **14** epandrial complex, posterior view **15** phallic complex, ventral view **16** phallic complex, lateral view. Scale bar: 0.5 mm.

##### Diagnosis.

Frons with a brown median stripe extending from anterior margin to ocellar triangle; gena with broad brown stripe. Acrostichal setulae in 4 rows. Epandrium with a pair of dorsoapical processes in lateral view and a pair of lateral processes on anterior margin; hypandrium, gonopod, and phallus confluent together. Phallus with 2 pairs of lateral processes and a pair of median processes apically. Phallapodeme longer than phallus.

##### Description.

**Male.** Body length 3.7 mm.

***Head*** (Fig. 9) yellow. Face with an angular hump on middle of basal half; parafacial with sparse short hairs, with a black spot between eye and antennal bases, and with 5 long setae extending to gena. Frons ~1.3× longer than wide and parallel-sided, with a brown median stripe extending from anterior margin to ocellar triangle, and frons with short setulae on anterior half; ocellar triangle grayish black, ocellar setae very small, hair-like; fronto-orbital setae missing. Gena with broad brown stripe, ~1/2 height of eye. Antenna yellow, first flagellomere and arista missing. Proboscis yellow with white and black setulae, and with a pair of irregular lateral spots apically; palpus yellow with black setulae.

***Thorax*** (Fig. 11) brownish yellow, with grayish-white pruinescence. Mesonotum with 4 brown stripes extending to tip of scutellum. 0+3 dorsocentral setae, anteriormost dorsocentral seta away from scutal suture; acrostichal setulae in 4 rows; a pair of prescutellar setae, all setae on thorax missing. Dorsal margin of anepisternum and katepisternum pale yellow. One anepisternal seta, 1 katepisternal seta. ***Legs*** yellow, fore tarsomeres and hind legs missing, mid tarsomeres 4 and 5 brown. Fore femur with 7 posterodorsal setae and 7 posteroventral setae, fore tibia with a long dorsal preapical seta and a short apicoventral seta. Mid tibia with a strong dorsal preapical seta and an apicoventral seta. Hind femur with a weak preapical anterodorsal seta; hind tibia with a long dorsal preapical seta and a short apicoventral seta. ***Wing*** pale brown along anterior margin, a brown spot on each of the crossveins r-m and dm-cu; subcostal cell brown but pale brown apically. Haltere pale yellow.

***Abdomen*** (Fig. 12) yellow, tergites 2–6 blackish brown on posterior margin but yellow laterally. ***Male genitalia*** (Figs 13–16): syntergosternite confluent with epandrium, broad dorsally and narrow ventrally. Epandrium with a pair of long black conical dorsoapical processes in lateral view, with a pair of lateral processes on anterior margin. Surstylus situated in ventral angle and small, ventral margin with setae, hypandrium V-shaped, disconnected in the middle and with 2 pairs of inner processes apically. Gonopod short and thick, extending to both sides; hypandrium, gonopod and phallus confluent together. Phallus with 2 pairs of different lateral processes and a pair of median processes apically; median processes claviform in lateral view. Phallus deeply concave apically, phallapodeme claviform, longer than phallus.

**Female.** Unknown.

##### Remarks.

This new species is very similar to *Luzonomyzasinica* from China (Hainan) and Thailand in the body markings, wing type, and surstylus, but it can be separated from the latter by the brown mid tarsomeres 4 and 5 and the 2 pairs of apical processes of the phallus. In *Luzonomyzasinica*, the mid tarsi are yellow and the phallus is not bifurcated apically.

##### Distribution.

China (Yunnan).

#### ﻿ Luzonomyza serrata

Taxon classificationAnimaliaDipteraLauxaniidae

Li & Yang sp. nov.

7E9D92DC-B8D0-5F85-BA3B-0C8277F59474

http://zoobank.org/2820D475-A55B-49B3-8C3E-1D6318626B66

Figures 17–21 , 22–25

##### Type material.

***Holotype*.** ♂ (CAUC), China, Yunnan Province, Xishuangbanna Dai Autonomous Prefecture, Menglun Zhiwuyuan; 21°55'12"N, 101°16'20"E; 580 m; 22 Apr 2007; Wenliang Li leg. ***Paratype*.** 1 ♂(CAUC), China, Yunnan Province, Xishuangbanna Dai Autonomous Prefecture, Menglun No. 55 area; 21°56'5"N, 101°14'48"E; 540 m; 23 Apr 2007; Hui Dong leg.

**Figures 17–21.**
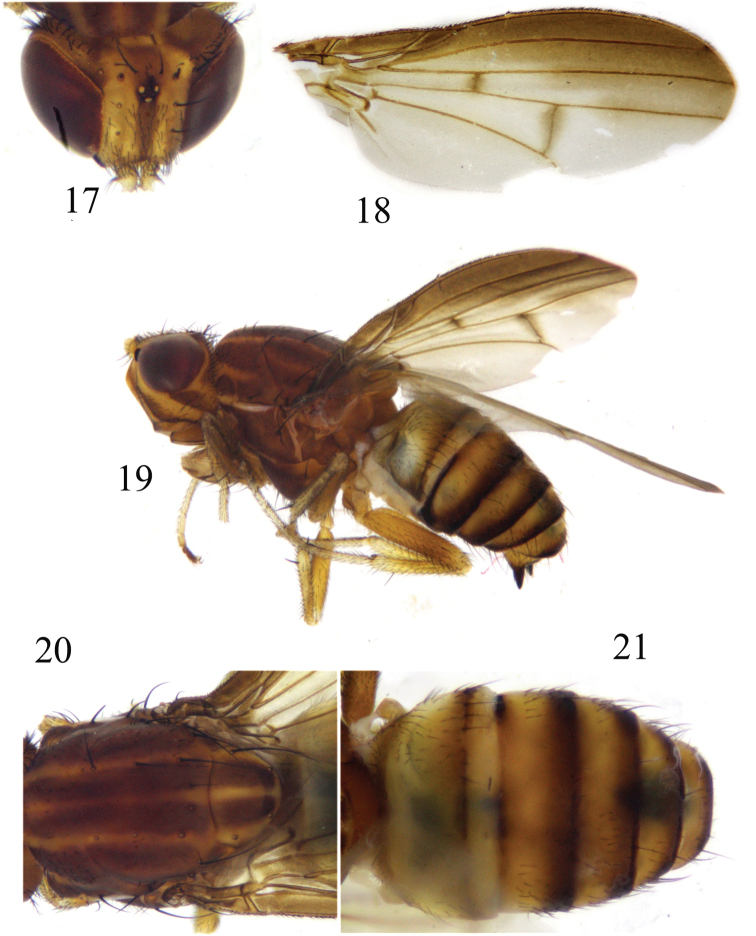
*Luzonomyzaserrata* sp. nov. Male **17** head, anterior view **18** wing **19** habitus, lateral view **20** thorax, dorsal view **21** abdomen, dorsal view.

##### Etymology.

Latin, *serrata*, referring to the serrated sides of the phallus.

**Figures 22–25.**
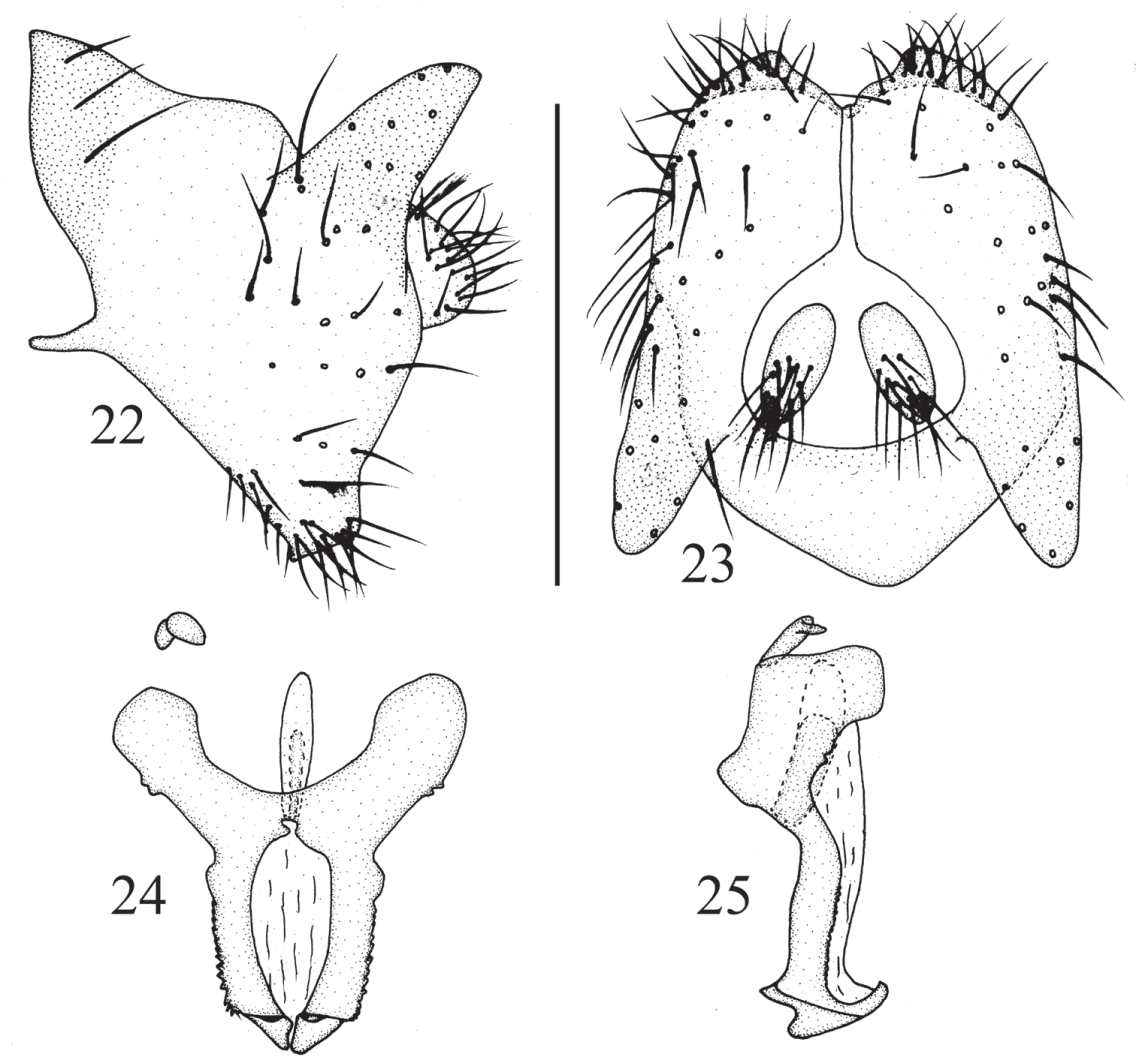
*Luzonomyzaserrata* sp. nov. Male **22** syntergosternite and epandrium, lateral view **23** epandrial complex, posterior view **24** phallic complex, ventral view **25** phallic complex, lateral view. Scale bar: 0.5 mm.

##### Diagnosis.

Face with 2 brown longitudinal stripes, gena with apical half of inner margin and ventral margin brown. Proboscis apically with a pair of Y-shaped brown spots. Mesonotum with 4 brown stripes extending to tip of scutellum. *Legs* pale yellow, fore femur with 7 posteroventral setae. Gonopod indistinct; both sides of phallus serrated. Phallus indistinct concave apically.

##### Description.

**Male.** Body length 3.4–4.0 mm, wing length 3.5–3.8 mm.

***Head*** (Fig. 17) yellow. Face with an angular hump on middle of basal half and 2 brown longitudinal stripes; parafacial with sparse short hairs and with 4 long setae extending to gena, with a black spot between eye and antennal bases, gena with apical half of inner margin and ventral margin brown. Frons ~1.4× longer than wide and parallel-sided, with a brown median stripe extending from anterior margin to ocellar triangle and with short setulae on anterior half; ocellar triangle grayish black, ocellar setae very small, hair-like; anterior fronto-orbital seta reclinate, shorter than the posterior one. Gena ~half height of eye and with broad brown stripe. Antenna yellow, first flagellomere and arista missing. Proboscis yellow with white and black setulae, and apically with a pair of Y-shaped brown spots; palpus yellow with black setulae.

***Thorax*** (Fig. 20) brownish yellow, with grayish-white pruinescence. Mesonotum with 4 brown stripes extending to tip of scutellum, occupying most of scutellum. 0+3 dorsocentral setae, anteriormost dorsocentral seta away from scutal suture; acrostichal setulae in 4 rows; a pair of prescutellar setae, all setae on thorax missing. Dorsal margin of anepisternum and katepisternum yellow. One anepisternal seta, 1 katepisternal seta. ***Legs*** pale yellow, fore femur and basal half of tibia brown, tarsomeres 4 and 5 pale brown, mid and hind femora and tibiae yellow but pale yellow apically. Fore femur with 7 posterodorsal setae and 7 posteroventral setae, fore tibia with a long dorsal preapical seta and a short apicoventral seta. Mid tibia with a strong dorsal preapical seta and an apicoventral seta. Hind femur with a weak preapical anterodorsal seta; hind tibia with a long dorsal preapical seta and a short apicoventral seta. ***Wing*** brown along costal margin, extending to M_1_, a brown spot on each of the crossveins r-m and dm-cu; subcostal cell brown; costa with 2^nd^ (between R_1_ and R_2+3_), 3^rd^ (between R_2+3_ and R_4+5_), and 4^th^ (between R_4+5_ and M_1_) sections in ratios of 9.0: 1.6: 1.2; r-m beyond middle of discal cell; ultimate and penultimate sections of M_1_ in ratios of 5.9: 3.0; ultimate section of CuA_1_ ~1/5 of penultimate. Haltere pale yellow.

***Abdomen*** (Fig. 21) yellow, tergites 2–6 blackish brown on posterior margin. ***Male genitalia*** (Figs 22–25): syntergosternite confluent with epandrium, broad dorsally and narrow ventrally. Epandrium with a pair of long black conical dorsoapical processes in lateral view, with a pair of lateral processes on anterior margin. Surstylus absent, ventral margin with setae, hypandrium V-shaped, with a pair of lateral acute processes. Gonopod indistinct; hypandrium, gonopod and phallus confluent together. Both sides of phallus serrated, with a dorsal process apically in lateral view. Phallus indistinct concave apically. Phallapodeme claviform, shorter than phallus.

**Female.** Unknown.

##### Remarks.

This new species is very similar to *Luzonomyzapseudoforficula* from Thailand in the body markings, wing type, and leg color, but it can be separated from the latter by the yellow antenna and the phallus being serrated on both sides. In *Luzonomyzapseudoforficula*, the antenna is black, and the phallus is not serrated in ventral view.

##### Distribution.

China (Yunnan).

#### ﻿ Luzonomyza vittifacies

Taxon classificationAnimaliaDipteraLauxaniidae

Li & Yang sp. nov.

01813EEC-440A-55CF-9B45-8498BCBA848A

http://zoobank.org/E092BDD5-2FD7-4705-A2B6-E57085A69696

Figures 26–30 , 31–34

##### Type material.

***Holotype*.** ♂ (CAUC), China, Yunnan Province, Xishuangbanna Dai Autonomous Prefecture, Mengla County, yaoqu township; 21°43'32"N, 101°32'37"E; 780 m; 26 Apr 2007; Wenliang Li leg.

**Figures 26–30.**
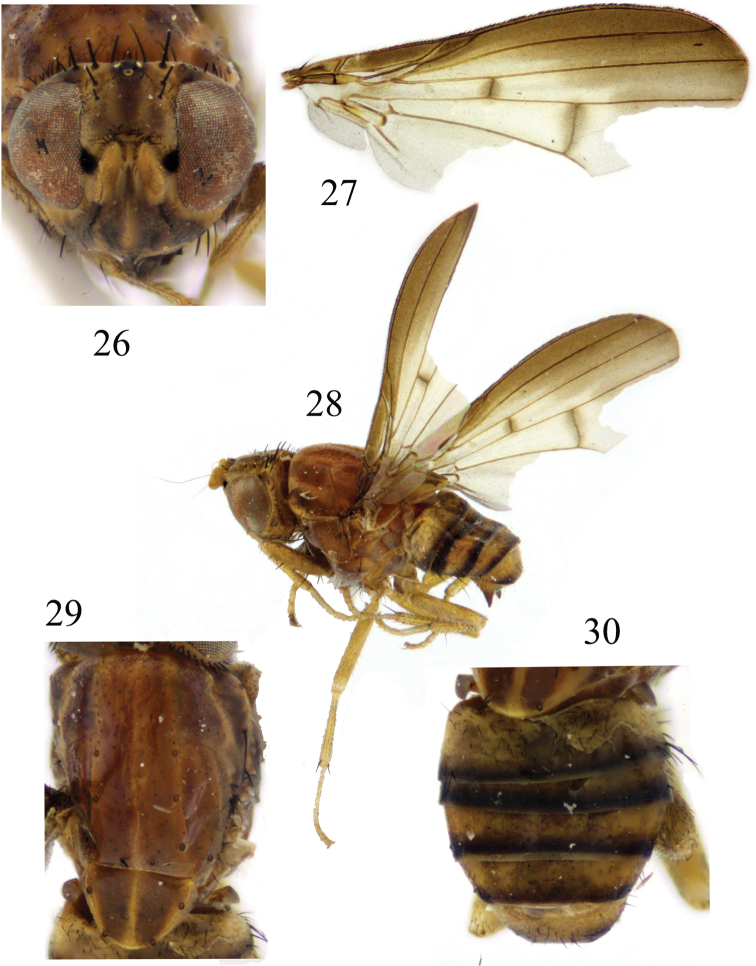
*Luzonomyzavittifacies* sp. nov. Male **26** head, anterior view **27** wing **28** habitus, lateral view **29** thorax, dorsal view **30** abdomen, dorsal view.

##### Etymology.

This epithet is an adjectival combination of the Latin adjective *vitti* (vittate) and noun *facies* (face), referring to the face with brown longitudinal stripes.

**Figures 31–34.**
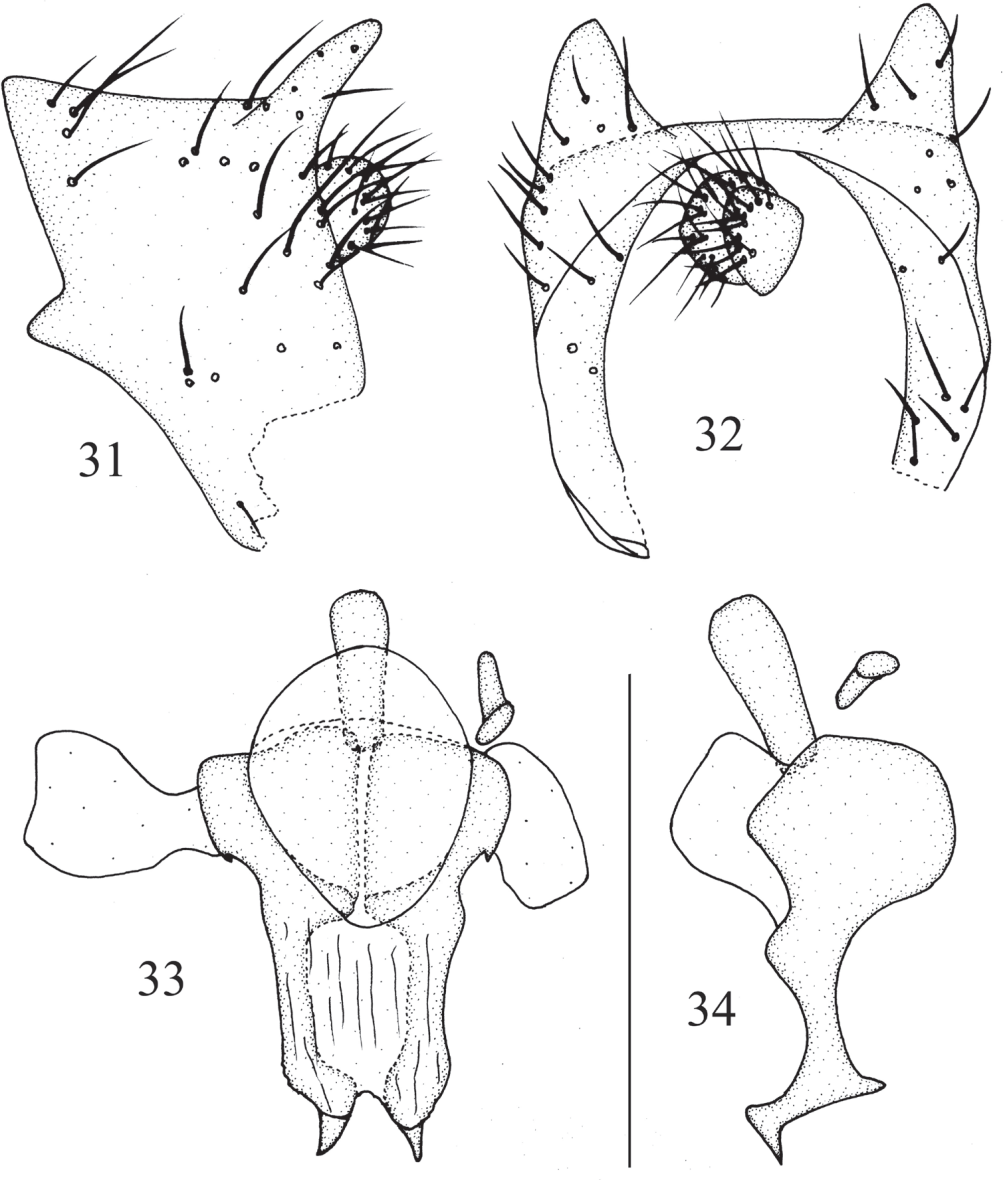
*Luzonomyzavittifacies* sp. nov. Male **31** syntergosternite and epandrium, lateral view **32** epandrial complex, posterior view **33** phallic complex, ventral view **34** phallic complex, lateral view. Scale bar: 0.5 mm.

##### Diagnosis.

Head yellow; face with a pair of brown stripes extending from antennal base and confluent on ventral margin. Thorax brownish yellow with grayish-white pruinescence; legs yellow, fore tarsomeres 4 and 5 brown. Abdomen yellow, tergites 2–6 black on posterior margin but tergite 6 yellow posteromedially. *Male genitalia*: hypandrium broad, membranous; pregonite confluent with hypandrium; phallus with a pair of lateral acute processes near base in ventral view, apical concave V-shaped, with a dorsal acute subapical process in lateral view, apex acute and curved dorsally.

##### Description.

**Male.** Body length 3.2 mm, wing length 3.4 mm.

***Head*** (Fig. 26) yellow. Face brownish yellow, with a pair of brown longitudinal stripes extending from antennal bases and confluent on ventral margin, face with an angular hump on middle of basal half; inner margin of parafacial with a brown stripe, broadening towards gena, with a black round spot between eye and antennal bases, parafacial with sparse short setulae, and 6 short black setae extending from parafacial ventral corner to gena. Frons as long as wide and parallel-sided, with a blackish-brown median line extending from anterior margin to ocellar triangle, and with short setulae on anterior half, denser on anterior margin; ocellar triangle grayish black; ocellar setae very small, hair-like, anterior fronto-orbital seta (situated at middle of fronto-orbital plate) reclinate, shorter than the posterior one. Gena ~1/3 height of eye and with broad brown stripe. Antenna yellow, first flagellomere brownish yellow and rounded apically, ~1.5× longer than high; arista brown, pubescent. Proboscis mostly yellow except blackish brown at tip, with white and black setulae; palpus yellow with black setulae.

***Thorax*** (Fig. 29) brownish yellow, with grayish-white pruinescence. Mesonotum with 4 broad brown stripes, 2 brown median stripes extending to tip of scutellum; 0+3 dorsocentral setae, anteriormost dorsocentral seta away from scutal suture; acrostichal setulae in 4 rows; a pair of prescutellar setae, missing. One anepisternal seta, 1 katepisternal seta. ***Legs*** mostly yellow, fore tarsomeres 4 and 5 brown. Fore femur with 8 posterodorsal setae and 6 posteroventral setae, fore tibia with a long dorsal preapical seta and a short apicoventral seta. Mid tibia with a strong dorsal preapical seta and an apicoventral seta. Hind tibia with a long dorsal preapical seta and a short apicoventral seta. ***Wing*** brown along costal margin extending to M_1_ and with hyaline spots, a brown spot on each of the crossveins r-m and dm-cu; subcostal cell brown; costa with 2^nd^ (between R_1_ and R_2+3_), 3^rd^ (between R_2+3_ and R_4+5_) and 4^th^ (between R_4+5_ and M_1_) sections in ratios of 7.1: 1.4: 1.2; r-m beyond middle of discal cell; ultimate and penultimate sections of M_1_ in ratios of 4.9: 2.5; ultimate section of CuA_1_ ~1/5 of penultimate. Haltere yellow.

***Abdomen*** (Fig. 30) yellow, tergites 2–6 black on posterior margin, but tergite 6 yellow posteromedially. ***Male genitalia*** (Figs 31–34): syntergosternite confluent with epandrium. Epandrium with a pair of black long conical dorsoapical processes in lateral view. Surstylus broken, hypandrium broad and membranous. Gonopod confluent with hypandrium, phallus consisting of a pair of sclerites in ventral view and a pair of lateral acute processes near base, with V-shaped apical concavity, broad basally and narrow apically in lateral view, with a dorsal acute process subapically, apex acute and curved dorsally. Phallapodeme claviform in ventral view. Ejaculatory apodeme bent.

**Female.** Unknown.

##### Remarks.

This new species is very similar to *Luzonomyzagaimarii* from China (Yunnan) in the wing spots and the thoracic and abdominal spots, but it can be separated from the latter by the pair of brown stripes on the face that extend down from antennal base and are confluent on ventral margin; by the 4 rows of acrostichal setulae; by r-m extending beyond middle of discal cell; by the absence of a lateral concavity in the hypandrium; and by the phallus being acute and curved dorsally in lateral view. In *Luzonomyzagaimarii*, the face has no spot; the acrostichal setulae are arranged in 2 rows; crossvein r-m is in the middle of the discal cell; the hypandrium has a lateral concavity; and the phallus is not curved dorsally in lateral view.

##### Distribution.

China (Yunnan).

## ﻿Discussion

This study increases the number of known *Luzonomyza* species in the world to 13. Seven species occur in China, accounting for more than half of the total species worldwide. Except for *L.sinica* in Hainan Province, the other six species are all found in Yunnan Province. There may be several reasons for the high number of species in Yunnan. One reason may be the location in the southwest of China, which has diverse climate types and a unique geographical location with suitable climate conditions. It has been considered to be a hotspot of global biodiversity for many years, and its fauna has attracted much attention. Another important reason may be that the seemingly more limited distribution of this genus may be the result of insufficient sampling outside Yunnan. There are still many areas in China with rich biological biodiversity that remain poorly investigated. We believe that the species diversity of the genus *Luzonomyza* in China may be underestimated, and there may be new species in other areas, so further investigations are needed.

## Supplementary Material

XML Treatment for Luzonomyza brevis

XML Treatment for Luzonomyza honghensis

XML Treatment for Luzonomyza serrata

XML Treatment for Luzonomyza vittifacies
